# Deep Reinforcement Learning for Autonomous Underwater Navigation: A Comparative Study with DWA and Digital Twin Validation

**DOI:** 10.3390/s26072179

**Published:** 2026-04-01

**Authors:** Zamirddine Mari, Mohamad Motasem Nawaf, Pierre Drap

**Affiliations:** 1 DGA Techniques Navales, Direction Générale de l’Armement, 83000 Toulon, France; zamirddine.mari@intradef.gouv.fr; 2 Laboratoire d’Informatique et des Systèmes, CNRS UMR 7020, Aix-Marseille University, 13013 Marseille, France; pierre.drap@image4d.org

**Keywords:** reinforcement learning, autonomous underwater vehicle, obstacle avoidance, dynamic window approach, 3D reconstruction

## Abstract

Autonomous navigation in underwater environments is challenged by the absence of GPS, degraded visibility, and submerged obstacles. This article investigates these issues using the BlueROV2, an open platform for scientific experimentation. We propose a deep reinforcement learning approach based on the Proximal Policy Optimization (PPO) algorithm, using an observation space that combines target-oriented navigation information, a virtual occupancy grid, and raycasting along the boundaries of the operational area. This information is encoded into a high-dimensional observation space of 84 dimensions, providing the agent with comprehensive local and global situational awareness. The learned policy is compared against a reference deterministic kinematic planner, the *Dynamic Window Approach* (DWA), a robust baseline for obstacle avoidance. The evaluation is conducted in a realistic simulation environment and complemented by validation on a physical BlueROV2 supervised by a 3D digital twin of the test site, reducing risks associated with real-world experimentation. The results show that the PPO policy consistently outperforms DWA in highly cluttered environments, notably thanks to better local adaptation and reduced collisions. Finally, experiments demonstrate the transferability of the learned behavior from simulation to the real world, confirming the relevance of deep RL for autonomous navigation in underwater robotics.

## 1. Introduction

The autonomy of underwater vehicles has become a major challenge for the exploration, monitoring, and inspection of marine environments. Underwater robots—whether fully autonomous AUVs or semi-autonomous ROVs—play an increasingly important role in scientific observation, bathymetric mapping, offshore infrastructure inspection, maintenance operations, and security and defense missions. The ability of these vehicles to navigate reliably and safely in complex, deep, or remote environments directly affects the quality, cost, and efficiency of such operations. In this context, improving autonomous navigation capabilities is essential to reducing dependence on teleoperation, extending mission duration, and increasing operational safety.

However, underwater environments impose severe constraints on autonomous navigation. The absence of GPS, heavily degraded visibility, hydrodynamic disturbances, and the presence of static obstacles (terrain features, rocks, wrecks, infrastructure) and dynamic obstacles (marine life, moving vessels) make trajectory planning difficult and uncertain. Moreover, the diversity of operational contexts—from cluttered coastal zones to industrial structures and complex natural environments—requires real-time adaptation capabilities. Ensuring safe behaviors therefore demands strategies capable of handling obstacle avoidance, stability control, and continuous local situational awareness. Finally, real-world experimental validation is costly and risky, which reinforces the importance of advanced simulation and digital twins for training and evaluating autonomous systems under realistic yet controlled conditions.

A robust way to tackle these challenges is to rely on open and modular experimental platforms that allow researchers to explore, test, and compare different autonomous navigation strategies in constrained environments. Among these platforms, the BlueROV2 has emerged as a reference vehicle for scientific research and algorithm development. Its open architecture, relatively low cost, and extensible software ecosystem make it an ideal testbed for the study of advanced navigation techniques, particularly those based on reinforcement learning. In this context, recent works have focused on three main axes: (i) navigation and obstacle avoidance approaches based on RL; (ii) research specifically involving the BlueROV2 as an experimental platform; and (iii) contributions leveraging digital twins to bridge simulation and reality in complex underwater environments.

### 1.1. Autonomous Navigation and Obstacle Avoidance Using Reinforcement Learning

The application of reinforcement learning (RL) to underwater navigation is an active and rapidly growing research area. Bhopale et al. [1] propose an RL-based obstacle avoidance technique enabling AUVs to learn effective behaviors without requiring a detailed dynamic model. Eweda and ElNaggar [2] provide an extensive review of the challenges faced by AUVs in dynamic environments, highlighting the ability of deep RL to produce robust policies despite disturbances and energy constraints.

Several methodological developments have since emerged to improve the stability and efficiency of learning. Marchel et al. [3] demonstrate the benefits of *Curriculum Learning* for training an RL agent to navigate environments of progressively increasing difficulty. Liu et al. [4] explore offline RL for obstacle avoidance in an underactuated AUV, while Manderson et al. [5] leverage a goal-conditioned architecture to learn visual behaviors directly from images.

These contributions reflect a growing interest in RL as a solution for advanced autonomy. However, to rigorously evaluate its real-world potential, it is essential to compare RL-based methods with established deterministic baselines.

### 1.2. Comparison Between Deterministic Approaches and Learning-Based Methods: DWA as a Baseline Against RL

In the mobile robotics literature, the *Dynamic Window Approach* (DWA) [6] remains one of the most widely used reactive obstacle avoidance algorithms. Its simplicity, computational efficiency, and native integration into ROS make it a standard baseline for assessing the advantages of RL policies.

Several studies have undertaken direct comparisons between RL and DWA. Patel et al. [7] show that DRL policies can outperform DWA in environments populated with moving obstacles, notably by reducing collisions and dynamic constraint violations. Arce et al. [8] conduct a structured comparative evaluation including DWA, TEB, CADRL, and an SAC agent, observing that RL agents generally surpass deterministic methods in complex environments. Yeom et al. [9] demonstrate that a DRL controller produces more efficient and smoother trajectories than DWA in a wheeled mobile robot scenario.

These comparative studies indicate that RL approaches tend to offer superior performance in dynamic, cluttered, or unstructured environments—an especially valuable property for underwater systems operating in unpredictable conditions.

### 1.3. Research on the BlueROV2 Platform

In parallel with methodological advances, several works specifically focus on the BlueROV2 platform. Wilby et al. [10] introduce a modified open-source variant called *Makobot*, optimized for autonomous missions. Willners [11] demonstrates the feasibility of transforming commercial ROVs such as the BlueROV2 into low-cost AUVs by adding onboard navigation and control capabilities.

However, despite its growing popularity as a research platform, few studies investigate the use of RL for obstacle avoidance on the BlueROV2. This gap highlights the relevance of developing and evaluating learning-based policies on this accessible and widely adopted platform.

### 1.4. Digital Twins and Underwater Modeling

Recent advances in simulation and digital twins have also strengthened validation capabilities in underwater robotics. Scaradozzi et al. [12] and Lambertini et al. [13] show that digital twin architectures enable fine-grained modeling of the robot and its environment, facilitating supervision and planning. Adetunji et al. [14] demonstrate that such systems improve teleoperation in complex scenarios.

These hybrid simulation–reality approaches are essential tools for training and testing RL policies prior to real-world deployment, helping to reduce risks and costs.

### 1.5. Positioning and Contributions

The reviewed literature demonstrates the relevance of RL for underwater obstacle avoidance while emphasizing the importance of comparing it with established deterministic methods such as DWA to rigorously assess its benefits. At the same time, the BlueROV2 has become a preferred experimental platform, and digital twins now offer powerful tools to secure and accelerate the transition from simulation to reality.

The present work lies at the intersection of these three research directions. It offers a controlled validation of a PPO policy for autonomous navigation of a BlueROV2, leveraging a realistic 3D environment capable of accurately simulating obstacles and local interactions. This approach enables the safe and reproducible evaluation of an RL agent’s ability to surpass a robust deterministic baseline such as DWA.

While PPO is an established algorithm, the novelty of this work lies in the integrated “System-of-Systems” architecture, specifically, the synchronization of a high-fidelity photogrammetric model with a real-time USBL-linked digital twin for “hardware-in-the-loop” safety validation, providing a reproducible pipeline for risky underwater RL deployments.

This positioning represents a significant step: it establishes the feasibility of RL-based autonomous control on a real underwater vehicle and demonstrates the value of coupling simulation with a physical robot. Building on this foundation, future work will extend these contributions toward integrating real perception sensors (video, sonar) and conducting trials in underwater environments featuring authentic obstacles and more diverse conditions. The long-term objective is to advance toward fully autonomous, robust, and deployable real-world underwater navigation.

## 2. Materials and Methods

**Figure 1:**
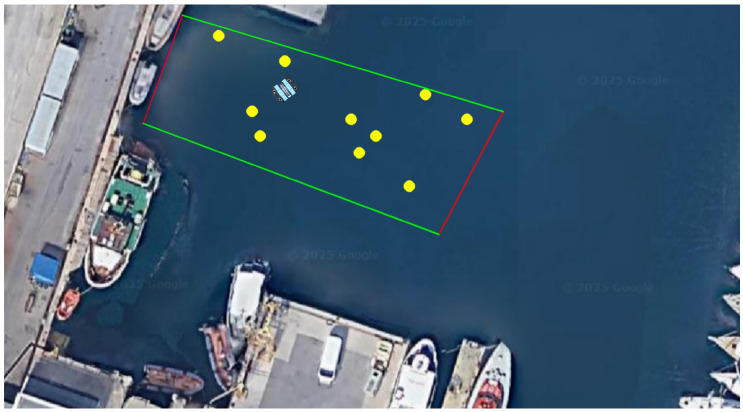
A screenshot showing the random placement of obstacles (yellow dots) within the zone (delimited by the straight lines, with red lines denoting starting and target positions) used for both RL and DWA tests.

This section describes the methodological approach adopted to study the autonomous navigation of the BlueROV2 underwater vehicle in a partially unknown environment containing submerged obstacles. Specifically, the navigation scenario illustrated in Figure 1 involves moving the vehicle at a fixed depth from one side of a rectangular area to the other while avoiding approximately ten static obstacles.

The objective is to evaluate the ability of a deep reinforcement learning approach to ensure safe and efficient navigation in direct comparison with a classical motion planner. Two paradigms are therefore considered: (i) a deterministic method based on the *Dynamic Window Approach* (DWA) and (ii) a learning-based method using *reinforcement learning* (RL) with the *Proximal Policy Optimization* (PPO) algorithm [15].

The first subsection introduces the comparative framework between these two approaches, while the second formalizes the problem as a Markov Decision Process (MDP), which serves as the basis for training the RL policy.

### 2.1. Comparative Framework of Navigation Strategies

We begin by establishing the comparative framework between the two studied methods by first presenting the theoretical foundations and implementation principles of the *Dynamic Window Approach* (DWA). The reinforcement learning approach, based on the PPO algorithm, is then introduced and detailed in the subsequent subsection.

1. Deterministic Approach: Dynamic Window Approach (DWA) DWA relies on a predictive evaluation of a discrete set of admissible kinematic commands for the vehicle. At each cycle, the algorithm considers a family of actions parameterized by an angular variation and a traversable distance, defining a finite set of possible future configurations.
  Predictive Model: For each candidate action, the vehicle projects its future position:
    $$x^{\prime }=x+d\;\left(\begin{array}{c} \cos(\theta +\Delta \theta )\\ \sin(\theta +\Delta \theta ) \end{array}\right),$$
    where x is the current position, θ the orientation, Δθ the candidate angular variation, and *d* the forward distance.
  Distance to the Goal: The attraction cost toward the goal, defined as the geometric center of the exit gate, is
    $$C_{\mathrm{goal}}\left(x^{\prime }\right)=∥x^{\prime }-g∥,$$
    where g denotes the target point.
  Obstacle Clearance: For each predicted configuration, the minimum safety distance to obstacles is computed as
    $$\delta \left(x^{\prime }\right)=\min_{i}\left(∥x^{\prime }-o_{i}∥-r_{i}-r_{\mathrm{robot}}-m_{s}\right),$$
    where $o_{i}$ and $r_{i}$ are respectively the position and radius of obstacle *i*, $r_{\mathrm{robot}}$ is the robot’s safety radius, and $m_{s}$ is an additional safety margin. A configuration is rejected if $\delta (x^{\prime })\leq 0$. A normalized clearance score is defined as
    $$S_{\mathrm{clear}}\left(x^{\prime }\right)=\min\;\left(1,\frac{\delta (x^{\prime })}{D_{\max}}\right).$$
  Kinematic Progress: The forward progress is represented by the traveled distance:
    $$S_{\mathrm{prog}}=d.$$
  Global Cost Function: Each command is evaluated using a weighted combination:
    $$J\left(x^{\prime }\right)=-\alpha \;C_{\mathrm{goal}}\left(x^{\prime }\right)+\beta \;S_{\mathrm{clear}}\left(x^{\prime }\right)+\gamma \;S_{\mathrm{prog}},$$
    where (α,β,γ) modulate the relative influence of each criterion. The selected action is then
    $$\left(\Delta \theta^{⋆},d^{⋆}\right)=\arg\max_{\Delta \theta ,d}J\left(x^{\prime }\right).$$
  This fully reactive method is a standard in mobile robotics. However, its effectiveness strongly depends on parameter tuning and the accuracy of sensed data, which may limit performance in dense, noisy, or structurally complex environments.
2. Learning-Based Approach: Reinforcement Learning (RL): The choice of a learning-based method is justified by the non-convex nature of the underwater obstacle avoidance problem. Unlike deterministic methods that may fail in local minima, the PPO algorithm utilizes a stochastic policy to explore the high-dimensional state space (84 dimensions), systematically optimizing the trajectory through policy gradient updates based on the Advantage Actor–Critic framework.

The comparison between these two paradigms aims to determine to what extent a learned policy can rival a classical deterministic planner. The following subsection details the theoretical framework and implementation details of the reinforcement learning approach.

### 2.2. Reinforcement Learning Problem Formulation

The objective is to determine an optimal control policy that guides the vehicle toward its target while ensuring safe navigation.

This problem is formulated as a *Markov Decision Process* (MDP) [16], defined by the tuple 〈S,A,P,R〉, where

- S is the set of observable system states (robot position, heading, obstacle distances, etc.);
- A is the set of admissible actions, here corresponding to discrete heading variations;
- $P(s^{\prime }|s,a)$ is the transition probability from state *s* to state $s^{\prime }$ after executing action *a*;
- R(s,a) is the immediate reward measuring the quality of the action (progress toward the target, obstacle avoidance, etc.).

The goal of the agent is to maximize the cumulative return defined as

(1) $$G_{t}=\sum_{k=0}^{\infty }\gamma^{k}R_{t+k+1},$$

where 0≤γ<1 is the discount factor weighting future rewards. The agent therefore learns a stochastic policy π(a|s) that maximizes the expected return $E[G_{t}]$.

To solve this MDP, we use a deep reinforcement learning method based on the *Proximal Policy Optimization* (PPO) algorithm. PPO is a policy optimization method that improves training stability by constraining successive policy updates through a clipped objective:

(2) $$L^{\mathrm{CLIP}}\left(\theta \right)=E_{t}\left[\min\left(r_{t}\left(\theta \right){\hat{A}}_{t},\mathrm{clip}\left(r_{t}\left(\theta \right),1-\epsilon ,1+\epsilon \right){\hat{A}}_{t}\right)\right],$$

where $r_{t}\left(\theta \right)=\frac{\pi_{\theta }\left(a_{t}|s_{t}\right)}{\pi_{\theta_{\mathrm{old}}}\left(a_{t}|s_{t}\right)}$ is the probability ratio, ${\hat{A}}_{t}$ an estimate of the advantage, and ϵ a trust-region hyperparameter typically between 0.1 and 0.2. PPO is chosen for its robustness, ease of implementation, and strong performance in complex robotic navigation tasks [15].

In this context, the navigation task is treated as a constrained optimization problem where the objective is to find the optimal policy parameters $\theta^{*}$ that maximize the expected return:

(3) $$\theta^{*}=\arg\max_{\theta }E_{\tau ∼\pi_{\theta }}\left[\sum_{t=0}^{T}\gamma^{t}r\left(s_{t},a_{t}\right)\right]$$

subject to the safety constraints $C_{collision}\left(s_{t}\right)=0$ and boundary constraints $C_{area}\left(s_{t}\right)=1$. This transforms the “trial and error” nature of exploration into a structured stochastic search within a defined feasible action space.

### 2.3. Simulation Environment

The simulation environment serves as the training and evaluation framework for the BlueROV2 agent. The goal is to guide the vehicle from an initial position located on one side of a quadrilateral domain representing the workspace to a target position placed on the opposite side. This space contains submerged obstacles which are not pre-mapped in the agent’s state. The control policy must therefore steer the vehicle to the target while respecting spatial constraints and avoiding collisions.

To ensure a rigorous scientific framework, the navigation task is defined as a constrained optimization problem. The agent must maximize its objective function while adhering to the following operational and physical constraints:

- Kinematic Constraint: To respect the vehicle’s physical inertia and thruster limits, the heading variation Δθ is restricted to a maximum of $\pm 45^{\circ }$ per decision step.
- Safety Constraint (Hard): A safety radius $r_{safe}$ of 0.5 m is enforced around the vehicle; any penetration of this volume by an obstacle is classified as a terminal failure.
- Spatial Constraint: The vehicle is bounded by the x,y coordinates of the reconstructed photogrammetric model, representing the physical limits of the operational harbor zone.

These constraints transform the learning process from a simple “trial and error” exercise into a structured search for a feasible policy within a bounded control space.

#### 2.3.1. Global Frame Modeling

**Figure 2:**
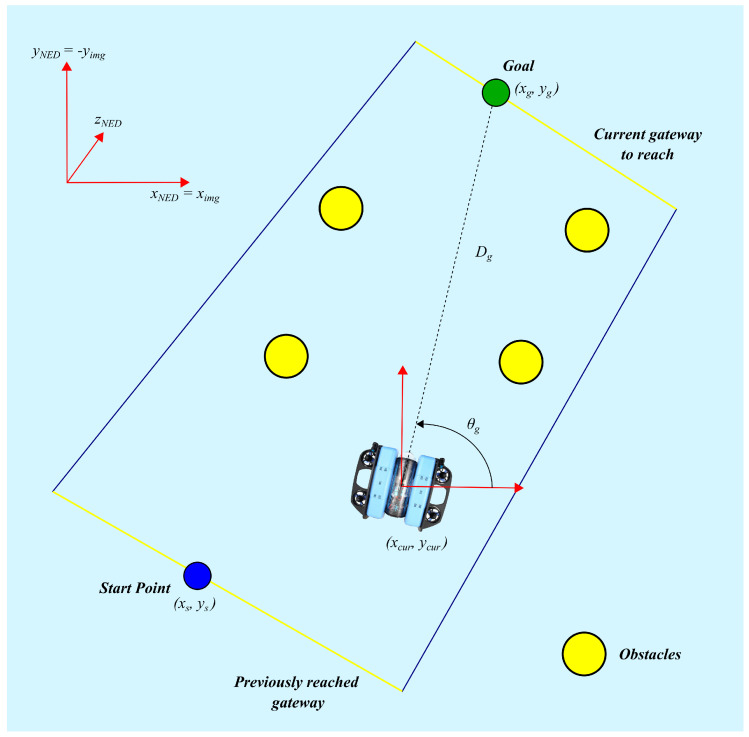
Diagram illustrating the interaction between the navigation agent and the simulated environment.

As illustrated in Figure 2, The environment is modeled using the conventional marine robotics *North*–*East*–*Down* (*NED*) coordinate system, where $x_{\mathrm{NED}}$ represents the North coordinate, $y_{\mathrm{NED}}$ the East coordinate, and $z_{\mathrm{NED}}$ the Down coordinate. As the vehicle operates at a fixed depth, trajectory planning is restricted to the horizontal plane defined by $\left(x_{\mathrm{NED}},y_{\mathrm{NED}}\right)$.

#### 2.3.2. Kinematic State and Autonomous Navigation Objective

The kinematic state of the BlueROV2 is defined by

(4) $$s_{\mathrm{NED}}\left(t\right)=\left[\begin{array}{c} x_{\mathrm{NED}}\left(t\right)\\ y_{\mathrm{NED}}\left(t\right)\\ \theta (t) \end{array}\right],$$

where $\left(x_{\mathrm{NED}},y_{\mathrm{NED}}\right)$ is the position and θ(t) the orientation with respect to the positive East axis $y_{\mathrm{NED}}$. The planar dynamics are

(5) $$\left\{\begin{array}{c} {\dot{x}}_{\mathrm{NED}}\left(t\right)=v\left(t\right)\sin\theta \left(t\right),\\ {\dot{y}}_{\mathrm{NED}}\left(t\right)=v\left(t\right)\cos\theta \left(t\right), \end{array}\right.$$

where v(t) is the translational velocity. Autonomous navigation aims to generate a trajectory Γ(t) from the initial state $s(t_{0})$ to the final state $s(t_{f})$, while ensuring

(6) $$\Gamma \left(t\right)\subset Q\setminus \overset{n}{\underset{i=1}{⋃}}O_{i},\;\forall t\in \left[t_{0},t_{f}\right],$$

where Q is the working domain and $\left\{O_{1},\ldots ,O_{n}\right\}$ the set of submerged obstacles.

### 2.4. Observation Space of the Autonomous Navigation Agent

The observation space defines all variables perceived by the agent at each navigation step. In our case, the agent receives an observation vector that integrates both target-oriented navigation information and local obstacle detection.

#### 2.4.1. Distance and Direction to the Goal

Let (x(t),y(t)) be the vehicle position and $\left(x_{g},y_{g}\right)$ the target (gateway) center. The normalized distance to the goal is

(7) $$d_{g}\left(t\right)=\frac{∥\left(x\left(t\right),y\left(t\right)\right)-\left(x_{g},y_{g}\right)∥}{d_{\max}},$$

with $d_{\max}$ a normalization constant corresponding to the maximum possible distance. The relative angle between the vehicle heading θ(t) and the direction to the goal is

(8) $$\Delta \theta_{g}\left(t\right)=\arctan2\left(y_{g}-y\left(t\right),x_{g}-x\left(t\right)\right)-\theta \left(t\right),$$

mapped to [−π,π] and normalized to [0,1]:

(9) $${\tilde{\theta }}_{g}\left(t\right)=\frac{|\Delta \theta_{g}\left(t\right)|}{\pi }.$$

Thus, the first observation component is

(10) $$o_{1}\left(t\right)=\left[\begin{array}{c} d_{g}\left(t\right)\\ {\tilde{\theta }}_{g}\left(t\right) \end{array}\right].$$

#### 2.4.2. Obstacle Detection via Occupancy Grid

**Figure 3:**
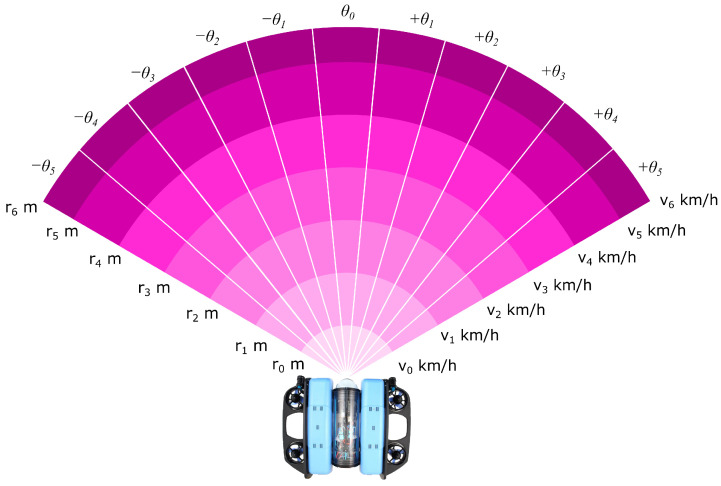
Virtual polar occupancy grid used for obstacle detection.

Obstacle perception is based on a virtual polar occupancy grid inspired by a forward-looking sonar. We consider discrete velocity values V, detection distances D, and angular directions A, as illustrated in Figure 3. Each triplet $\left(v_{i},d_{j},\alpha_{k}\right)$ defines a grid cell indicating whether a trajectory following this configuration would lead to a collision. The occupancy indicator is

(11) $$O_{j,k}=\left\{\begin{array}{cc} 1 & \mathrm{if}\;\mathrm{an}\;\mathrm{obstacle}\;\mathrm{is}\;\mathrm{detected}\;\mathrm{in}\;\mathrm{sector}\;(d_{j},\alpha_{k}),\\ 0 & \mathrm{otherwise}. \end{array}\right.$$

associated observation vector is

(12) $$o_{2}\left(t\right)={\left[O_{j,k}\left(t\right)\right]}_{j=1..n,\;k=1..p}.$$

While this virtual grid provides a robust baseline for policy training, it omits real-world sonar artifacts such as multi-path interference and acoustic noise. Future work will involve replacing virtual raycasting with raw sonar point clouds to better capture the perception noise, latency, and sensor failure modes encountered in real underwater sensing.

#### 2.4.3. Raycasting for Workspace Boundary Awareness

**Figure 4:**
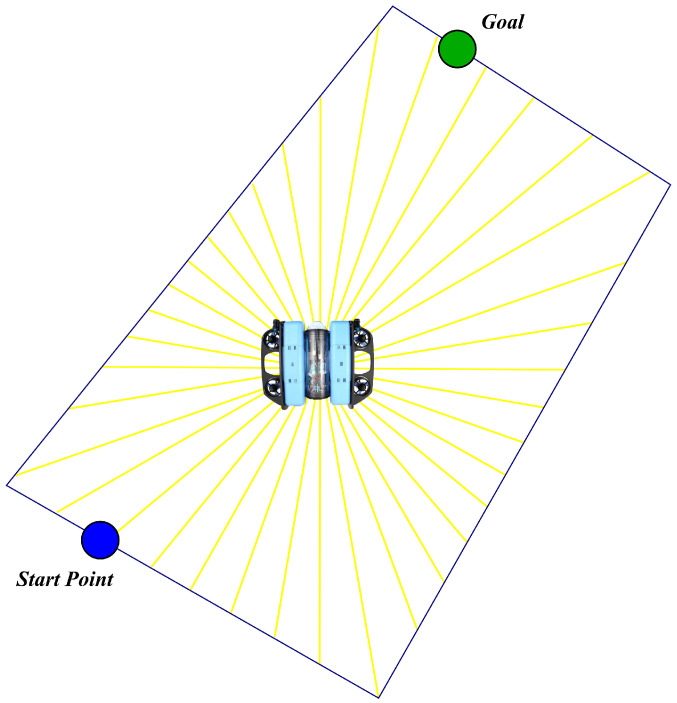
Virtual rays used to ensure that the vehicle remains inside the workspace.

To ensure that the agent remains within the quadrilateral workspace, a set of rays $\left\{r_{1},\ldots ,r_{q}\right\}$ is cast from the vehicle in predefined angular directions, as illustrated in Figure 4. The normalized length of each ray is

(13) $$\rho_{\ell }\left(t\right)=\frac{\ell_{\min}\left(r_{\ell }\right)}{\ell_{\max}},$$

where $\ell_{\min}\left(r_{\ell }\right)$ is the distance from the ray origin to the first intersection with the quadrilateral and $\ell_{\max}$ the maximum ray length. The resulting observation vector is

(14) $$o_{3}\left(t\right)={\left[\rho_{\ell }\left(t\right)\right]}_{\ell =1..q}.$$

#### 2.4.4. Final Observation Vector

The full observation vector is constructed by flattening and concatenating the component vectors:

(15) $$o\left(t\right)=\left[o_{1}\left(t\right),o_{2}\left(t\right),o_{3}\left(t\right)\right].$$

This observation space provides the agent with all necessary information to navigate toward the target while avoiding obstacles and respecting workspace constraints.

**Figure 5:**
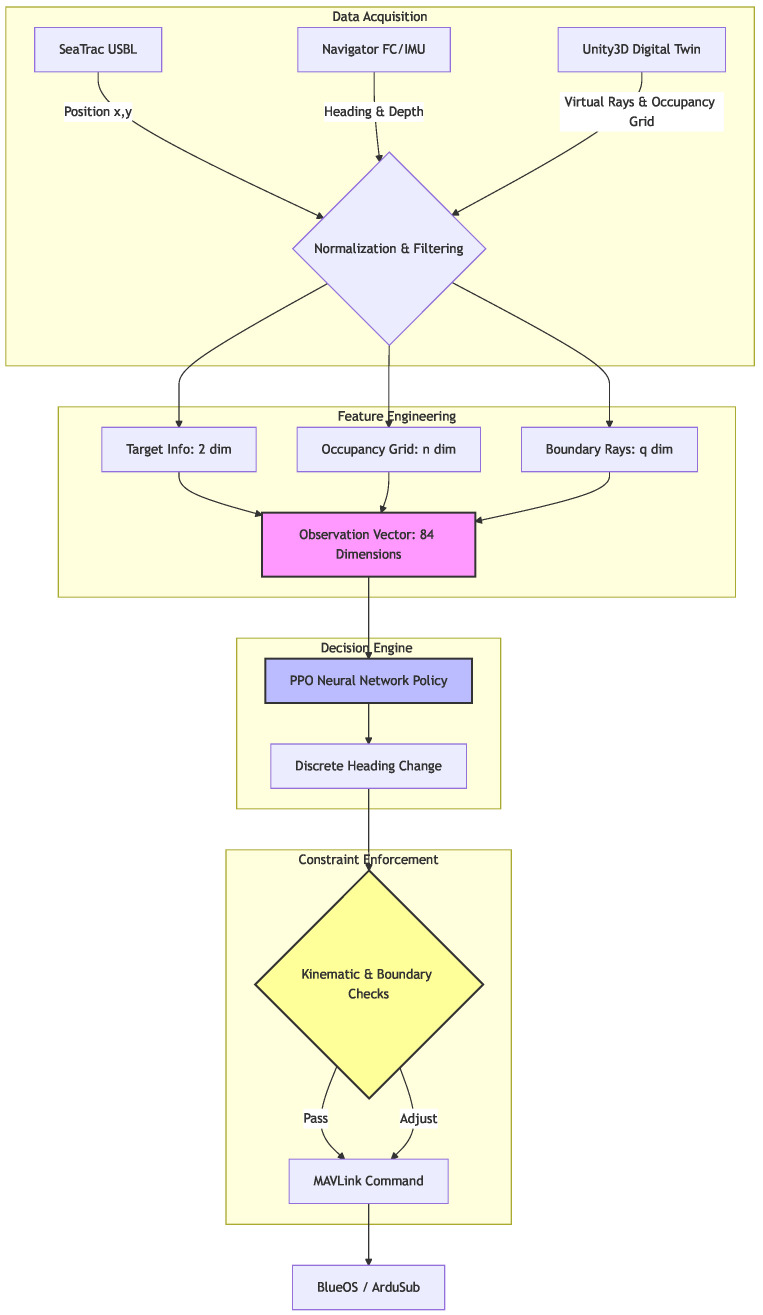
The perception–action pipeline of the autonomous navigation agent. This diagram illustrates the transition from raw sensor data to a structured 84-dimensional observation space, the stochastic optimization process within the PPO policy, and the final deterministic constraint enforcement layer that ensures kinematic and spatial safety before command execution.

The functional architecture of the navigation system is synthesized in Figure 5. This diagram maps the operational flow from the acquisition of multi-modal sensor data to the generation of heading commands, highlighting the structured integration of the 84-dimensional observation space with the stochastic decision engine and the subsequent safety filter.

### 2.5. Action Space

The action space corresponds to all discrete control inputs that the agent can take to modify its trajectory. For the BlueROV2, the action selected at each step corresponds to a relative adjustment of the vehicle’s heading.

We define a finite set of N=7 actions corresponding to discrete angular variations within the range [−π/4,+π/4]. The set of admissible heading changes is defined as

(16) $$A=\left\{a_{0},a_{1},\ldots ,a_{6}\right\},\;\Delta \theta_{a}\in \left\{-\frac{\pi }{4},-\frac{\pi }{6},-\frac{\pi }{12},0,\frac{\pi }{12},\frac{\pi }{6},\frac{\pi }{4}\right\}.$$

**Figure 6:**
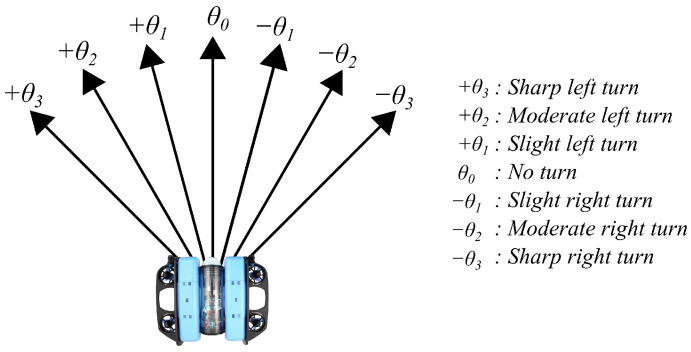
Discretization of actions into elementary angular variations.

Thus, $a_{0}$ corresponds to a sharp left turn ($-45^{\circ }$), while $a_{6}$ corresponds to a sharp right turn ($+45^{\circ }$). The central action $a_{3}$ maintains the current heading, as illustrated in Figure 6.

Let the kinematic state at time *t* be

(17) $$s\left(t\right)=\left[\begin{array}{c} x(t)\\ y(t)\\ \theta (t) \end{array}\right].$$

When an action *a* is applied, the orientation becomes

(18) $$\theta \left(t+1\right)=\theta \left(t\right)+\Delta \theta_{a}\;\mathrm{mod}\;2\pi ,$$

and the position updates with constant speed *v* and step size Δd:

(19) $$\left[\begin{array}{c} x(t+1)\\ y(t+1) \end{array}\right]=\left[\begin{array}{c} x(t)+\Delta d\cos(\theta (t+1))\\ y(t)+\Delta d\sin(\theta (t+1)) \end{array}\right].$$

This discrete kinematic model allows the agent to implicitly learn the correlation between actions and obstacle clearance, enabling the policy to select actions that ensure safety while progressing toward the target.

#### Reward Shaping

The reward function is constructed from local progress updates, intermediate milestones, and terminal events (success or failure), which are finally aggregated into a single instantaneous reward value.

*Local Progress:* Normalized progress between entry and exit of the workspace corridor is
  $$p_{t}=\mathrm{progress}\left(x_{t}\right)\in \left[0,1\right],\;\Delta p_{t}=p_{t}-p_{t-1}.$$

Strictly positive progress is rewarded via

$$r_{t}^{\mathrm{prog}}=b_{\mathrm{prog}}\;p_{t},$$

where $b_{\mathrm{prog}}$ is a scaling coefficient. If the agent does not progress ($\Delta p_{t}\leq 0$), no reward is given and no penalty is applied in the proposed formulation of the step() function.

*Intermediate Milestones:* To reinforce long-term structure, three intermediate milestones are defined:

$$p_{t}\in \left\{0.25,\;0.50,\;0.75\right\}.$$

When a milestone is crossed between two time steps,

$$r_{t}^{\mathrm{milestone}}=\left\{\begin{array}{cc} B_{1/4}, & \mathrm{if}\;0.25\;\mathrm{is}\;\mathrm{crossed},\\ B_{1/2}, & \mathrm{if}\;0.50\;\mathrm{is}\;\mathrm{crossed},\\ B_{3/4}, & \mathrm{if}\;0.75\;\mathrm{is}\;\mathrm{crossed}. \end{array}\right.$$

Thus, for $\Delta p_{t}>0$, the progress reward is

$$r_{t}^{\mathrm{progress}}=r_{t}^{\mathrm{prog}}+r_{t}^{\mathrm{milestone}}.$$

*Obstacle Avoidance and Safety (Terminal Events):* Two terminal negative events produce a penalty and immediately end the episode:

$$\mathrm{collision}\left(x_{t}\right)\;\mathrm{or}\;\neg \mathrm{inside}\_\mathrm{track}\left(x_{t}\right)\;\Rightarrow \;r_{t}=B_{\mathrm{fail}}<0.$$

*Terminal Success Reward:* Reaching the exit gate terminates the episode with a positive reward:

$$r^{\mathrm{success}}=B_{\mathrm{succ}}.$$

*Final Instantaneous Reward:* The reward returned at each time step is

$$r_{t}=\left\{\begin{array}{cc} B_{\mathrm{fail}}, & \mathrm{if}\;\mathrm{collision}\;\mathrm{or}\;\mathrm{track}\;\mathrm{exit},\\ B_{\mathrm{succ}}, & \mathrm{if}\;\mathrm{gateway}\;\mathrm{reached},\\ r_{t}^{\mathrm{progress}}, & \mathrm{if}\;\Delta p_{t}>0,\\ 0, & \mathrm{otherwise}. \end{array}\right.$$

The reward coefficients ($B_{succ},B_{fail},B_{prog}$) were determined through an empirical calibration process. The success reward was set an order of magnitude higher than the maximum possible cumulative progress reward to prevent the agent from “circling” to accumulate small gains. Similarly, the collision penalty $B_{fail}$ was weighted to ensure that avoiding an obstacle is always prioritized over a risky shortcut toward a milestone.

## 3. Experimental Results

This section presents the results obtained during the training, evaluation, and validation of the deep reinforcement learning model developed for the autonomous navigation of the BlueROV2. The analysis includes: (i) a description of the training parameters, (ii) the evolution of the agent’s training performance, (iii) a quantitative comparison with the DWA algorithm, and (iv) validation on a real robot at sea.

### 3.1. Training Parameters

Below are the training parameters used to train the model with Ray version 2.49.2 and its RLlib framework dedicated to reinforcement learning.

#### 3.1.1. Model Architecture

The model is based on a PPO architecture using an MLP network that processes an 84-dimensional state vector and includes:

- Three fully connected layers of size [128,128,128];
- Tanh activation functions for the main layers;
- Observation normalization;
- Separate actor–critic heads for PPO;
- No convolutional layers (vector-based observations only).

#### 3.1.2. Training Hyperparameters

The main hyperparameters used to train the autonomous navigation agent are summarized in Table 1.

## 4. Analysis of Training Performance

**Figure 7:**
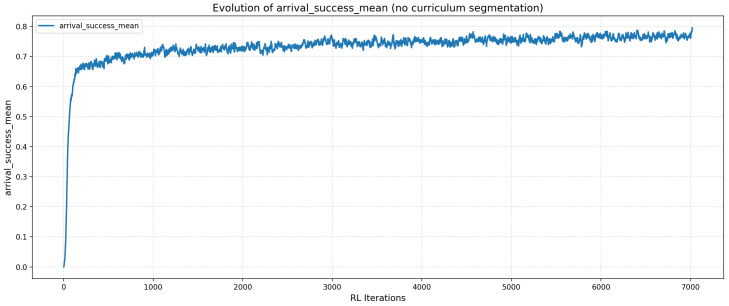
Average arrival success rate per iteration for the proposed RL agent.

**Figure 8:**
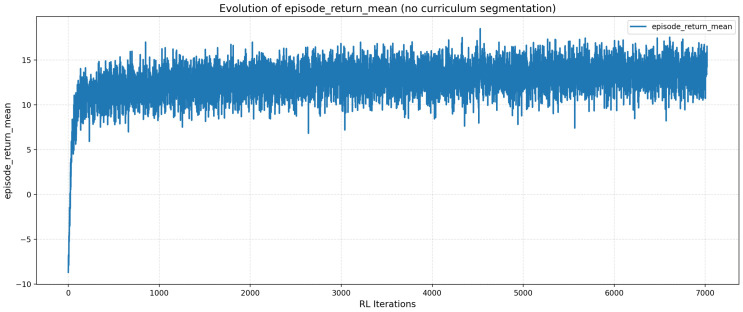
Progression of the cumulative reward per episode for the RL agent.

The training performance of the autonomous navigation agent is evaluated using two primary metrics: (i) the success rate (*arrival*_*success*_*mean*), which measures the frequency of reaching the final waypoint without collision and (ii) the average episode return (*episode*_*return*_*mean*), representing the cumulative reward. The evolution of these metrics over 7k training iterations is illustrated in Figure 7 and Figure 8, providing insight into the policy’s convergence and stability.

### 4.1. Evolution of the Success Rate

The success rate curve shown in Figure 7 exhibits a globally stable progression throughout the training phase. The statistical summary of this performance is presented in Table 2.

These results demonstrate that the agent quickly achieves and maintains a success rate between 73% and 75%. The initial minimum (0.0004) corresponds to the exploratory phase prior to policy stabilization. Ultimately, the progression indicates that the agent effectively learns to reach the target gateway while navigating up to ten obstacles, resulting in a well-converged and robust policy.

### 4.2. Evolution of the Cumulative Reward

The evolution of the average reward per episode, illustrated in Figure 8, follows a trend consistent with the improvement in the success rate. The summary statistics for the training process are presented in Table 3.

The negative minimum corresponds to the early exploratory phase, while the interquartile range (from 11.76 to 14.03) shows limited dispersion, indicating a stable policy centered around high reward values. The coherence between the increase in reward and the success rate confirms that the agent effectively learns to avoid collisions while optimizing its trajectory toward the target gateway.

The strong correlation between the increase in average reward and the success rate confirms that the agent effectively learns to avoid obstacles while optimizing its trajectory. Furthermore, the high average return suggests that the agent consistently reaches the intermediate milestones, validating the effectiveness of the reward shaping strategy in guiding the vehicle efficiently toward the target gateway.

### 4.3. Global Interpretation

The joint analysis of the success rate and cumulative reward highlights an efficient, high-performing, and stable navigation policy. The key performance indicators (KPIs) of the trained agent are synthesized in Table 4, illustrating the policy’s operational robustness. The scientific validity of the resulting policy is assessed not only by the success rate but also through specific Performance Indicators (KPIs): trajectory efficiency (ratio of actual path length to Euclidean distance) and safety margin (average distance to obstacles). These metrics provide a quantitative basis for evaluating the optimization beyond simple arrival success.

Ultimately, the training results reflect more than just numerical convergence; they represent a functional policy capable of navigating dense and randomized environments. The consistency of the success rate and the stability of the rewards suggest that the agent has internalized a robust strategy for obstacle avoidance rather than merely memorizing specific scenarios. This capacity for generalization is a critical prerequisite for the real-world deployment and comparison phases discussed in the following sections.

### 4.4. Comparison with the Reference Algorithm (DWA)

To assess the robustness and effectiveness of the proposed PPO model, we conducted a systematic comparison with the Dynamic Window Approach (DWA) benchmark. Both approaches were evaluated in a simulated environment containing ten obstacles, focusing on three key operational metrics: (i) success rate (reaching the target gateway), (ii) collision rate, and (iii) rate of workspace boundary violations (out-of-area).

The results, obtained over 100 evaluation episodes where obstacle configurations were identical for both algorithms to ensure statistical fairness, are presented in Table 5. The data indicates that the PPO model significantly outperforms DWA in cluttered environments. While DWA reaches the objective in only 8% of episodes, the PPO model achieves a success rate of 55%, representing a nearly seven-fold improvement. The collision rate is also drastically reduced with the learned policy (17% versus 76% for DWA). This suggests that while DWA often becomes trapped in local minima or fails to find a feasible path in high-density obstacle fields, the RL agent exhibits superior predictive capabilities and more robust obstacle negotiation.

Regarding workspace boundary violations, DWA records a lower rate (16%) compared to PPO (28%). This discrepancy is attributed to the inherent nature of the PPO policy, which prioritizes goal-reaching and collision avoidance through wider maneuvers. In highly constrained scenarios, the agent may opt for large-scale trajectory deviations that occasionally lead to workspace exits to avoid imminent collisions—a behavior that highlights the trade-off between local safety and global path planning.

Overall, these results demonstrate the clear superiority of the PPO-based approach in terms of navigation efficiency and collision mitigation in complex underwater scenarios, validating its suitability for autonomous maritime missions.

To ensure statistical relevance beyond high-density cases, the model was tested across varying obstacle distributions. As shown in Table 5, the PPO policy maintains a significant performance edge even as environmental complexity and obstacle density scale, demonstrating its generalized competence.

### 4.5. Interpretation of the Performance Gap Between Training and Evaluation

A discrepancy is observed between the average success rate during the training phase (approximately 74%) and the performance recorded during the 100-episode comparative benchmark (55%). This variation is anticipated and can be attributed to several methodological and environmental factors inherent to the transition from learning to inference.

To further clarify this gap, Table 6 summarizes the environmental complexity during both phases. The evaluation phase specifically used a “stress-test” configuration with 40% higher obstacle density than the training average, explaining the 19% performance drop.

First, the evaluation protocols differ in terms of task distribution. The training success rate is an average computed over more than 7000 iterations, encompassing a vast spectrum of randomized obstacle configurations. Conversely, the 100-episode PPO–DWA benchmark utilizes a static, high-density obstacle protocol designed to stress-test the algorithms. These evaluation scenarios often represent a “harder” subset of the distribution compared to the average training episode, which naturally leads to a lower absolute success rate.

Second, the transition from an evolving, stochastic policy during training to a fixed, deterministic policy for evaluation introduces a performance shift. During training, the agent utilizes exploration to navigate complex states; in the fixed evaluation phase, the agent relies solely on the learned mapping without the benefit of continuous weight updates. This phenomenon highlights a standard *distribution shift* between the training experiences and the specific test-set constraints.

Finally, the sample size of 100 episodes, while statistically significant for comparison, is subject to higher variance in highly stochastic underwater environments. In such cluttered domains, even a policy with high generalized competence may encounter a cluster of edge-case configurations that impede success within a limited sample window.

Despite this gap, the PPO model’s significant margin of superiority over the DWA baseline (55% vs. 8% success) confirms its superior generalization capabilities in unstructured environments. The performance difference is therefore interpreted not as a failure of the model, but as a rigorous reflection of the agent’s robustness when confronted with a higher difficulty threshold than that encountered on average during the learning phase.

### 4.6. Sea Experiments

**Figure 9:**
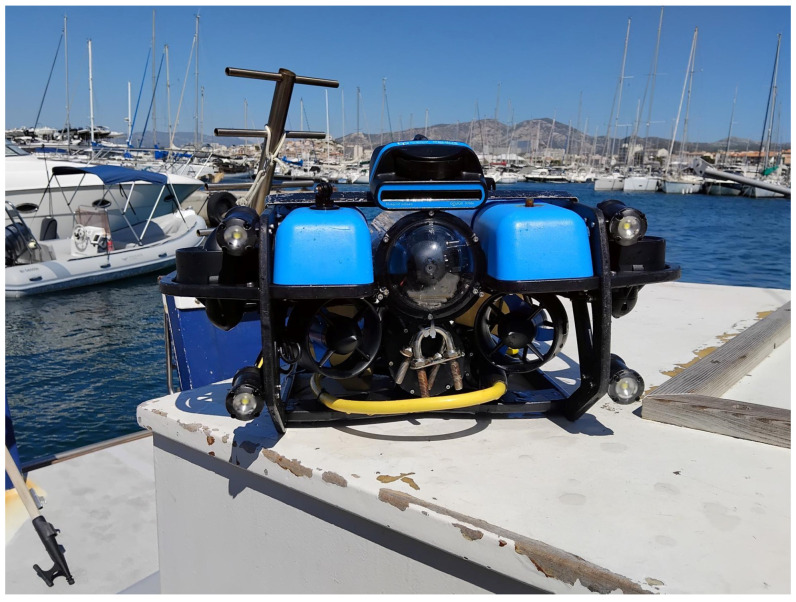
Photo of the BlueROV2 used during sea trials in the Pointe Rouge harbor (Marseille).

The sea trials conducted in the Pointe Rouge harbor (Marseille) were designed to validate the autonomous control policy on a physical *BlueROV2 Heavy* platform (Figure 9) in a real-world marine environment. The experiments focused on assessing the agent’s ability to navigate toward a target while avoiding fixed obstacles that were virtually rendered within the 3D digital twin environment. This “hardware-in-the-loop” safety approach allowed for the evaluation of the RL policy’s reactivity and stability without risking physical damage to the vehicle or the harbor infrastructure.

#### 4.6.1. Hardware Configuration

The experimental platform is based on the BlueROV2 Heavy configuration, equipped with an onboard Inertial Measurement Unit (IMU), a pressure-based depth sensor, and a forward-facing camera. Precise underwater localization is achieved using a SeaTrac USBL (Ultra-Short Baseline) acoustic positioning system by Blueprint Subsea [17], which provides sub-metric accuracy within the trial area. A surface control station is linked to the vehicle via a neutral buoyancy tether, facilitating low-latency, bidirectional communication for the transmission of real-time telemetry and the execution of the deep reinforcement learning (DRL) control commands.

#### 4.6.2. Software Architecture

Control of the BlueROV2 relies on a software stack built on MAVLink, providing the interface between the onboard hardware and the RL decision module. The RL module continuously receives vehicle states (estimated position, orientation, obstacle distances) and sends back the optimal control actions. This real-time perception–action loop ensures the reactivity required for obstacle avoidance while maintaining stable behavior.

#### 4.6.3. Software Architecture

**Figure 10:**
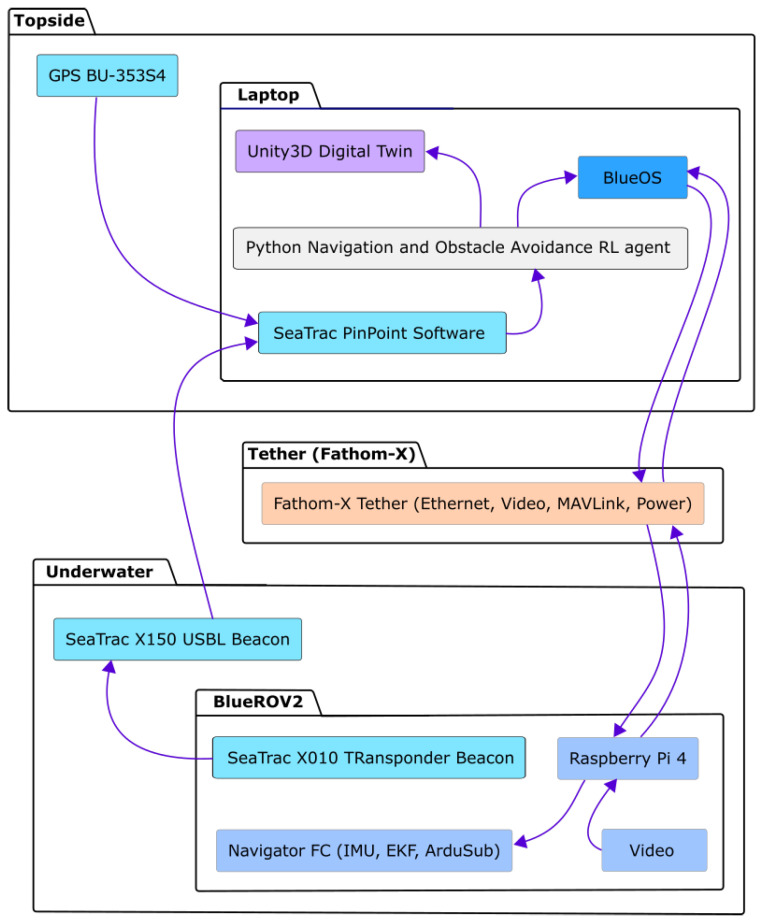
System architecture for sea trials: integration of hardware components (BlueROV2, SeaTrac USBL), communication protocols (MAVLink), and the digital twin environment for real-time monitoring.

The software architecture (Figure 10) is designed to bridge the high-level decision-making of the deep reinforcement learning (DRL) agent with the low-level hardware control of the BlueROV2. The control stack utilizes the MAVLink communication protocol, which serves as the primary interface between the ArduSub flight controller and the RL decision module. This module operates in a high-frequency perception–action loop: it continuously ingests processed vehicle states, including the USBL-derived position, orientation from the IMU, and virtual obstacle distances, and computes optimal heading adjustments. These adjustments are then translated into MAVLink commands and dispatched to the vehicle’s thrusters. This architecture ensures the real-time reactivity necessary for dynamic obstacle avoidance while maintaining the operational stability of the platform.

#### 4.6.4. Digital Twin and Visual Monitoring

**Figure 11:**
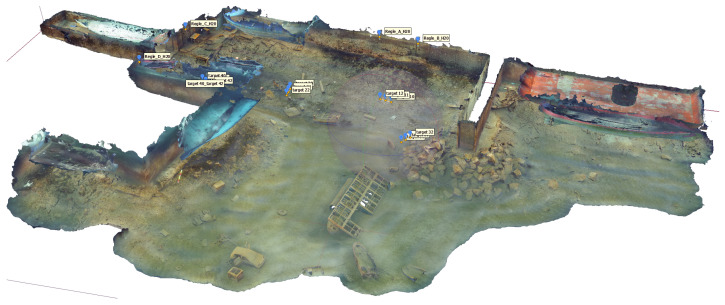
Photogrammetric 3D reconstruction of the underwater test site using Agisoft Metashape.

**Figure 12:**
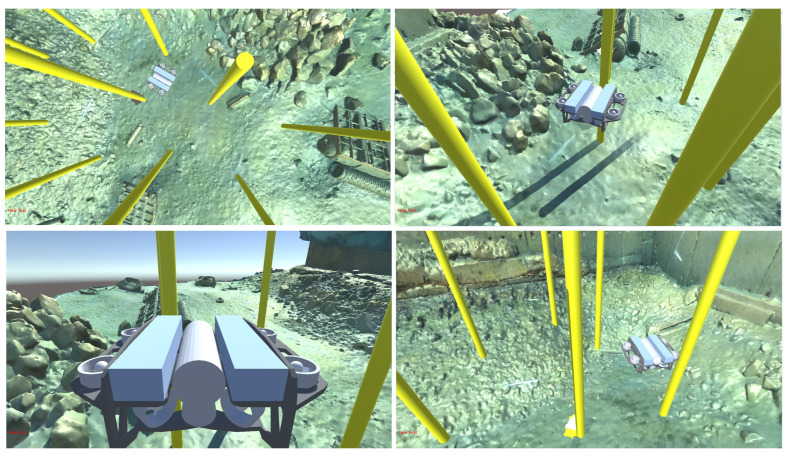
Unity3D environment visualizing the autonomous vehicle avatar within the high-fidelity photogrammetric model.

The foundation of the digital twin is a high-resolution 3D photogrammetric reconstruction of the underwater test site, as illustrated in Figure 11. The survey was conducted by divers who manually acquired a massive dataset comprising 26K images using an NIKON D800 (14 mm) camera, while ensuring comprehensive coverage and high redundancy. The photogrammetric processing is performed in Agisoft Metashape Professional. The resulting model provides a highly detailed representation of the operational area, covering approximately 2967 m^2^ with a ground resolution of 0.313 mm/pix. Accuracy was strictly maintained using six control scale bars, achieving a total RMS error of 3.2 mm. The final reconstructed Digital Elevation Model (DEM) and arbitrary 3D mesh (comprising over 4 million faces) were integrated into the Unity3D (2023.3.xxf1) engine to serve as the immersive virtual environment (Figure 12).

This digital twin is synchronized in real time with the vehicle’s position as estimated by the SeaTrac USBL acoustic system. This allows for the simultaneous visualization of the BlueROV2 avatar and its virtual camera feed within the digital environment. This simulation–reality coupling provides essential visual supervision and a quantitative basis for comparing real-world performance against simulated trajectories.

#### 4.6.5. Field Validation and Sim-to-Real Performance Analysis

The navigation experiments focused on reaching a predefined global target while navigating through a field of stationary obstacles. These obstacles were rendered exclusively within the 3D digital twin to ensure zero physical risk during the validation phase. Each experimental sequence successfully validated the end-to-end autonomous pipeline, encompassing state estimation, RL-based decision-making, and real-time motor execution on the physical platform.

**Figure 13:**
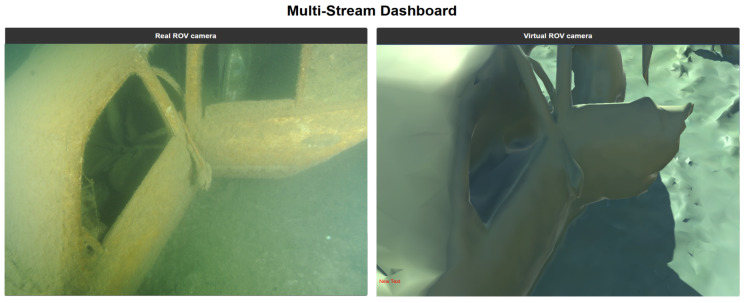
Real-time synchronization between the physical BlueROV2 camera feed and the digital twin’s virtual perspective.

The sea trials confirmed the system’s ability to maintain behavioral consistency during the Sim-to-Real transfer. The BlueROV2 followed trajectories that closely mirrored those predicted in the simulation environment. The real-time synchronization between the SeaTrac USBL data and the 3D avatar allowed for a direct qualitative and quantitative comparison of the camera feeds (Figure 13).

Quantitatively, the sea trials yielded a Mean Absolute Error (MAE) of 0.85 m between the planned RL trajectory and the USBL-tracked path. This error remains within the operational safety buffer, validating the policy’s practical stability and real-time reactivity in the presence of unmodeled disturbances.

Observed deviations between the planned and executed trajectories remained within acceptable operational bounds. These discrepancies are primarily attributed to stochastic noise inherent to acoustic positioning and the unmodeled hydrodynamic disturbances of the harbor environment. Despite these sources of aleatoric uncertainty, the PPO agent demonstrated robust goal-seeking behavior by consistently pursuing the target while maintaining a safe distance from virtual obstacles.

These results validate the feasibility of deploying an RL policy trained in a synthetic environment to control a BlueROV2 in a physical marine setting. The digital twin proved to be a critical tool for risk-free analysis and interpretation of autonomous behaviors. This functional milestone confirms the relevance of the proposed methodology as a foundation for future work involving real-time perception sensors and more complex, unstructured underwater terrains.

## 5. Discussion

The simulation results demonstrate that the PPO-based autonomous navigation architecture facilitates the acquisition of robust and adaptive control policies in densely cluttered environments. By leveraging a hybrid observation space that integrates target-oriented navigation cues, a virtual polar occupancy grid, and raycasting-derived geometric data, the RL agent evolves reactive behaviors that significantly surpass the capabilities of manually tuned kinematic cost functions. The direct comparison with the deterministic DWA baseline reveals a distinct performance advantage for reinforcement learning in high-complexity scenarios, confirming its utility for underwater robotics in irregular or constrained spatial domains.

Validation in real-world conditions, facilitated by the digital twin of the test site, provides critical evidence for successful Sim-to-Real transfer. The trajectories observed on the physical BlueROV2 platform exhibited high qualitative consistency with the simulated paths, despite the presence of environmental disturbances. Discrepancies between the virtual and physical deployments are primarily attributed to the stochastic noise of the USBL acoustic positioning system and the nonlinear hydrodynamic uncertainties inherent to the harbor environment. These findings underscore the feasibility of learning-based autonomous control while highlighting the importance of addressing the Sim-to-Real gap through high-fidelity modeling and robust state estimation in underwater settings.

### 5.1. Improving Localization Through Visual Relocalization in a Photogrammetric Model

Sea trials emphasize the critical role of robust localization in ensuring reliable autonomous control. Currently, the BlueROV2 reference trajectory relies primarily on USBL acoustic positioning, which is subject to accuracy fluctuations and stability issues depending on the site’s acoustic configuration, and whose high cost can limit accessibility for smaller robotic platforms. A promising alternative is to exploit the extensive image dataset used during the photogrammetric 3D reconstruction of the operational site.

Rather than correlating real-time frames with synthetic renders from the digital twin, this approach establishes a direct correspondence between the live feed from the BlueROV2 camera and the original source images used in the photogrammetry process, whose 6-DOF (degree-of-freedom) poses are known with high metric accuracy. This principle of visual relocalization within a database of *pre*-*oriented* images can significantly refine the estimation of the vehicle’s orientation and local 3D position. Technically, this would be implemented via a loosely coupled fusion architecture: a Kalman Filter (EKF) would use the USBL as a global reference to bound long-term drift, while the high-frequency 6-DOF poses derived from visual relocalization would provide the precision necessary for near-obstacle maneuvering.

The prospects of such an approach extend beyond remote supervision. In industrial or strategically sensitive sites that have been pre-modeled, visual alignment with a photogrammetric dataset could form the basis of a low-cost, GPS-denied local positioning system. By correlating high-resolution images from an autonomous drone with the established model, platforms lacking expensive acoustic sensor suites could achieve high-fidelity localization during inspection or surveillance missions.

This approach nevertheless presents significant challenges for real-time onboard deployment: the management of massive image datasets, the robustness of feature descriptors in turbid or low-visibility environments, variability in underwater illumination, and the high computational cost of image-to-database matching. These technical constraints, however, represent valuable opportunities for future research into lightweight visual-inertial odometry and descriptor matching for underwater robotics.

The accuracy of the digital twin significantly influences control performance; specifically, unmodeled hydrodynamic currents in the DT can lead to a “reality gap” where the RL agent overestimates its maneuverability. In the presence of unexpected obstacles, a lower-fidelity DT may result in aggressive policies that fail when faced with real-world water resistance or thruster latency.

### 5.2. General Perspectives

Taken together, the results position this work as a significant step toward underwater autonomy driven by learning-based policies. The consistency of the Sim-to-Real trajectory transfer, the operational feasibility of the integrated simulation–reality pipeline, and the superior performance over the DWA baseline in constrained environments constitute foundational milestones for more complex mission scenarios.

The identified future research directions include:

- Integrating real-time perception data (video, sonar) directly into the decision loop;
- Extending navigation from planar movement to full three-dimensional (6-DOF) control, acknowledging that while 2D is sufficient for floor inspections, scaling to general AUV missions requires accounting for pitch, heave, and vertical hydrodynamic coupling;
- Exploring *safe RL* constrained optimization to mitigate the higher rate of workspace boundary exits observed in PPO by treating boundaries as hard constraints within the policy update;
- Investigating multi-agent reinforcement learning for collaborative missions;
- Developing visual relocalization within high-fidelity photogrammetric models to reinforce vehicle positioning.

Ultimately, these avenues converge toward a more robust, multi-modal, and operational framework for autonomy, in which learning-based policies interact seamlessly with realistic 3D models, diverse sensors, and complex underwater environments.

## 6. Conclusions

This work presented an initial validation of autonomous navigation for a BlueROV2 underwater vehicle based on deep reinforcement learning. The problem was formulated as a Markov Decision Process (MDP) with a hybrid observation space combining target-oriented navigation data, a local environment representation through a virtual polar occupancy grid, and geometric perception via raycasting. Building on this formulation, a PPO policy was trained and evaluated within a 3D environment faithfully reproducing the physical constraints and obstacles of the operational domain.

The experiments demonstrate that the RL agent is capable of ensuring safe and efficient navigation in cluttered environments, surpassing the DWA planner in the most complex scenarios. Validation on a physical BlueROV2, enabled by a high-fidelity digital twin of the test site, confirms the successful Sim-to-Real transfer of the learned policy and establishes the feasibility of RL-based autonomous control in underwater settings.

This research represents a foundational step toward integrating learning-based policies into real-world underwater missions. By establishing a safe, reproducible framework linked to a physical platform, this milestone facilitates a second phase involving real-time perception sensors (video, sonar) and trials in unstructured natural environments. A particularly promising direction involves improving relative positioning through visual relocalization within pre-constructed photogrammetric models. Additional perspectives include extending navigation to 3D control, investigating *safe RL* methods to strengthen operational safety, and exploring multi-agent strategies for cooperative missions. Ultimately, these advances aim to bring RL-based autonomous navigation closer to robust, real-world deployment.

## Figures and Tables

**Table 1:** Main hyperparameters used for PPO training of the BLUEROV2 agent.

Hyperparameters	Values
Learning rate	2.5 × $10^{-4}$
Gamma (γ)	0.95
λ (GAE)	0.9
Clip parameter	0.3
KL target	0.01
Entropy coeff	0.0
VF loss coeff	1.0
VF clip param	10.0
Num epochs	30
Rollout fragment length	30
Train batch size	1950
Minibatch size	128

**Table 2:** Statistical summary of the arrival success rate during training.

Mean	Median	Minimum	Maximum
0.734	0.745	0.0004	0.796

**Table 3:** Statistical summary of the average episode return during training.

Mean	Median	Minimum	Maximum
12.77	12.91	−8.69	18.50

**Table 4:** Synthesis of the agent’s performance and scientific evaluation metrics.

Metric Attribute	Operational/Scientific Significance
Robust Success Rate (∼74%)	Reliable navigation in complex, cluttered environments.
High Average Reward	Optimized trajectory planning and milestone achievement.
Trajectory Efficiency	Optimization of path length relative to Euclidean distance.
Safety Margin	Quantification of average clearance from submerged obstacles.
Low Statistical Variability	Strong convergence and consistent decision-making.

**Table 5:** Quantitative performance comparison between the DWA benchmark and the proposed PPO model over 100 test episodes.

Navigation Method	Success Rate (%)	Collision Rate (%)	Boundary Exit (%)
DWA (Baseline)	8	76	16
PPO (Proposed)	55	17	28

**Table 6:** Comparison of environmental difficulty between Training and Evaluation phases.

Metric	Training (Avg.)	Evaluation (Stress-Test)
Obstacle Count	5–10	10 (Fixed)
Obstacle Density (obs/m^2^)	0.003	0.005
Average Success Rate	74%	55%

## Data Availability

Dataset available on request from the authors.

## References

[1] Bhopale P., Kazi F., Singh N. (2019). Reinforcement Learning Based Obstacle Avoidance for Autonomous Underwater Vehicle. J. Marine Sci. Appl..

[2] Eweda M.M., ElNaggar K. (2024). Reinforcement learning for autonomous underwater vehicles (AUVs): Navigating challenges in dynamic and energy-constrained environments. Robot. Integr. Manuf. Control.

[3] Marchel Ł., Kot R., Szymak P., Piskur P. (2025). Model-Based AUV Path Planning Using Curriculum Learning and Deep Reinforcement Learning on a Simplified Electronic Navigation Chart. Appl. Sci..

[4] Zhao J., Liu T., Huang J. (2024). Hybrid Offline Reinforcement Learning for Obstacle Avoidance in Autonomous Underwater Vehicles. Ships Offshore Struct..

[5] Manderson T., Chen Z., Williams S. (2020). Vision-Based Goal-Conditioned Policies for Underwater Navigation. arXiv.

[6] Fox D., Burgard W., Thrun S. (1997). The dynamic window approach to collision avoidance. IEEE Robot. Autom. Mag..

[7] Patel U., Kumar N.K.S., Sathyamoorthy A.J., Manocha D. (2021). DWA-RL: Dynamically Feasible Deep Reinforcement Learning Policy for Robot Navigation among Mobile Obstacles. Proceedings of the 2021 IEEE International Conference on Robotics and Automation (ICRA), Xi’an, China, 18 October 2021.

[8] Arce D., Solano J., Beltrán C. (2023). A Comparison Study between Traditional and Deep-Reinforcement-Learning-Based Algorithms for Indoor Autonomous Navigation in Dynamic Scenarios. Sensors.

[9] Yeom K. (2022). Deep Reinforcement Learning Based Autonomous Driving with Collision Free for Mobile Robots. Int. J. Mech. Eng. Robot. Res..

[10] Wilby A., Lo E. (2020). Low-Cost, Open-Source Hovering Autonomous Underwater Vehicle (HAUV) for Marine Robotics Research based on the BlueROV2. Proceedings of the 2020 IEEE/OES Autonomous Underwater Vehicles Symposium (AUV), St. Johns, NL, Canada, 30 November 2020.

[11] Willners J.S., Carlucho I., Łuczyński T., Katagiri S., Lemoine C., Roe J., Stephens D., Xu S., Carreno Y., Pairet È. (2021). From market-ready ROVs to low-cost AUVs. OCEANS 2021: San Diego–Porto.

[12] Scaradozzi D., Gioiello F., Ciuccoli N., Drap P. (2024). A Digital Twin Infrastructure for NGC of ROV during Inspection. Robotics.

[13] Lambertini A., Menghini M., Cimini J., Odetti A., Bruzzone G., Bibuli M., Mandanici E., Vittuari L., Castaldi P., Caccia M. (2022). Underwater Drone Architecture for Marine Digital Twin: Lessons Learned from SUSHI DROP Project. Sensors.

[14] Adetunji F.O., Ellis N., Koskinopoulou M., Carlucho I., Petillot Y.R. (2024). Digital Twins Below the Surface: Enhancing Underwater Teleoperation. arXiv.

[15] Schulman J., Wolski F., Dhariwal P., Radford A., Klimov O. (2017). Proximal Policy Optimization Algorithms. arXiv.

[16] Sutton R.S., Barto A.G. (2018). Reinforcement Learning: An Introduction.

[17] Blueprint Subsea SeaTrac Micro-USBL Tracking and Data Modem System. https://www.blueprintsubsea.com/seatrac.

